# Differences in checkpoint-inhibitor-induced hypophysitis: mono- versus combination therapy induced hypophysitis

**DOI:** 10.3389/fendo.2024.1400841

**Published:** 2024-07-29

**Authors:** Stephanie van der Leij, Karijn P.M. Suijkerbuijk, Medard F.M. van den Broek, Gerlof D. Valk, Jan Willem Dankbaar, Hanneke M. van Santen

**Affiliations:** ^1^ Department of Endocrinology, University Medical Center, Utrecht University, Utrecht, Netherlands; ^2^ Princess Máxima Center for Pediatric Oncology, Utrecht, Netherlands; ^3^ Department of Medical Oncology, University Medical Center, Utrecht University, Utrecht, Netherlands; ^4^ Department of Radiology, University Medical Center, Utrecht University, Utrecht, Netherlands; ^5^ Department of Pediatric Endocrinology, Wilhelmina Children’s Hospital, University Medical Center, Utrecht University, Utrecht, Netherlands

**Keywords:** IR-hypophysitis, treatment corticosteroids, immune checkpoint inhibitors, empty sella, immune therapy toxicity

## Abstract

**Objective:**

Immune checkpoint inhibitors (ICIs) are revolutionary in oncology but may cause immune-related (IR) side effects, such as hypophysitis. Treatment with anti-PD-(L)1, anti-CTLA-4 or anti-CLTA-4/PD-1 may induce hypophysitis, but little is known about the differences in clinical presentation or need for different treatment. We analyzed the differences of anti-PD-(L)1, anti-CTLA-4 and anti-CTLA-4/PD-1 induced hypophysitis

**Methods:**

retrospective analysis of 67 patients (27 anti-PD-(L)1, 6 anti-CLTA-4 and 34 anti-CTLA-4/PD-1 induced hypophysitis).

**Results:**

The median time between starting ICIs and IR-hypophysitis was longer after anti-PD(L)-1) therapy (22 weeks versus 11 and 14 weeks after anti-CTLA-4 and anti-CTLA-4/PD-1 therapy, respectively). The majority of patients (>90%), presented with atypical complaints such as fatigue, nausea, and muscle complaints. Headache, TSH or LH/FSH deficiency were more common in anti-CTLA-4 and anti-CLTA-4/PD-1 versus anti-PD-(L)1 induced hypophysitis (83% and 58% versus 8%, 67% and 41% versus 11%, and 83% and 48% versus 7%, respectively). Pituitary abnormalities on MRI (hypophysitis or secondary empty sella syndrome) were only seen in patients receiving anti-CTLA-4 or anti-CTLA-4/PD-1 therapy. Recovery from TSH, LH/FSH and ACTH deficiency was described in 92%, 70% and 0% of patients after a mean period of 14 and 104 days, respectively, and did not differ between patients who did or did not receive high-dose steroids.

**Conclusion:**

The clinical presentation of IR-hypophysitis varies depending on the type of ICIs. MRI abnormalities were only seen in anti-CTLA-4 or anti-CTLA-4/PD-1 induced hypophysitis. Endocrine recovery is seen for LH/FSH and TSH deficiency but not for ACTH deficiency, irrespective of the corticosteroid dose.

## Introduction

In recent years, the development of immune checkpoint inhibitors (ICIs) has contributed to a revolution in cancer treatment (1–4). ICIs are not only the first choice of treatment in advanced melanoma but are also widely used for several types of solid cancers. With the introduction of ICIs, a new spectrum of endocrine, immune-related adverse events (eIRAEs) are observed: immune-related (IR)-thyroiditis or hypothyroidism, IR-hypophysitis, IR-diabetes, and rarely IR-adrenal insufficiency (5–7).

Whereas primary autoimmune hypophysitis is rare - with a 1 in 9 million prevalence - IR-hypophysitis is far more common (8). The prevalence of IR-hypophysitis depends on the type of ICI: it is described in 9–10% of patients treated with anti-CTLA-4/PD-1 combination therapy, in 2–6% of patients with anti-CTLA-4 monotherapy and in 1% of patients with anti-PD(L)1 monotherapy (5, 6, 9).

Clinical symptoms of IR-hypophysitis can be vague, and its symptoms may overlap with symptoms often seen in patients with advanced cancer, which makes it harder to recognize and to diagnose in an early stage (5, 6). Clinical symptoms and the degree of hypopituitarism seem to differ between anti-PD-(L)1 induced hypophysitis and anti-CTLA-4 induced hypophysitis (9). Adrenocorticotropic hormone (ACTH) deficiency is the most common pituitary deficiency in patients with IR-hypophysitis (95–97%). In one study, the prevalence of thyroid-stimulating hormone (TSH) deficiency and luteinizing hormone (LH)/follicle-stimulating hormone (FSH) deficiency was found to be higher in anti-CTLA-4-induced hypophysitis than in anti-PD-(L)1-induced hypophysitis, 85% and 75% versus 4% and 13%, respectively (9). Recovery from TSH deficiency and LH/FSH deficiency is reported in 83%-85% of patients. However, recovery from ACTH deficiency is very rare (6, 9–11) Although recovery from TSH and LH/FSH is frequently reported, the time to recovery is unclear. It is also unclear whether combination therapy with anti-CTLA-4/PD-1 differs clinically from anti-PD-(L)1-induced hypophysitis. Unfortunately, no randomized trials on the optimal screening and management of IR-hypophysitis exist. European guidelines for screening and treatment of IR-hypophysitis differ significantly between endocrinologists and oncologists (See **Supplementary Material**). Although recommended treatment strategies for IR-hypophysitis due to anti-CTLA-4 or anti-PD-(L)1 are currently the same, it may be questioned whether this should be further differentiated based on its etiology. To gain a better understanding of the clinical picture of IR-hypophysitis, we aimed to describe and compare the clinical presentation, including MR imaging and course of anti-PD-(L)1, anti-CTLA-4 and anti-CTLA-4/PD-1-induced hypophysitis.

## Materials and methods

**Figure 1:**
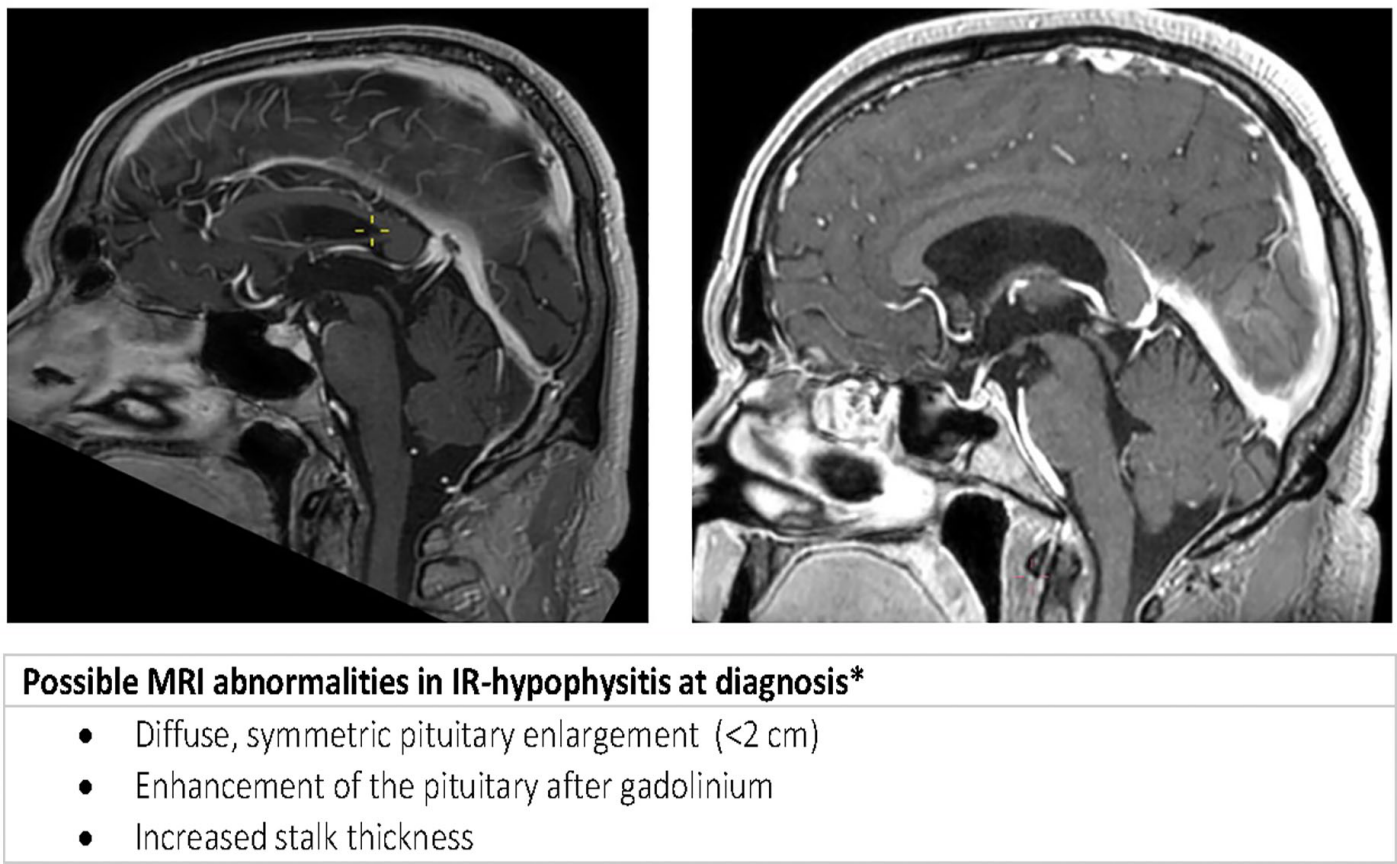
Sagittal MRI with radiological features of hypophysitis at presentation (left) and secondary empty sella during follow up (right).

All patients with suspected IR-hypophysitis referred to the Department of Endocrinology at the University Medical Center Utrecht between 2013 and 2022 were identified (n=97). The diagnostic criteria for IR-hypophysitis were clinical signs of hypophysitis in combination with biochemical evidence of anterior pituitary hormone deficiency and/or magnetic resonance imaging (MRI) suggestive of hypophysitis (9, 12) (**Figure 1**). Patients with prior treatment with systemic steroids were excluded (n=30).

Clinical data were obtained from the electronic patients’ files. They included sex, age, type of cancer, type of ICI, duration from start of ICIs to diagnosis of IR-hypophysitis, clinical symptoms of hypophysitis, laboratory results (sodium, glucose, ACTH, cortisol, TSH, FT4, prolactin, LH, FSH, estradiol or testosterone and insulin-like growth factor (IGF-1), imaging (MRI) and the presence of other immune related adverse events (IRAEs). The presence of IR-thyroiditis was based on the biochemical course of TSH and FT4 without the determination of thyroid peroxidase antibodies.

The following definitions for hypopituitarism were used: TSH deficiency was defined as a decreased concentration of TSH (<0.35 mIU/L) in combination with a low FT4 level (≤12 pmol/L) or a disproportionally low TSH level (0.35–7.0 mIU/L) with a decreased FT4 concentration (<10 pmol/L) (13). ACTH deficiency was defined as low random cortisol (<200 nmol/L) or a decreased morning cortisol (<250 nmol/L, without an increased ACTH concentration (4–60 ng/L), LH/FSH deficiency was defined as a low concentration of testosterone or estradiol with low or normal level of LH/FSH. Due to the absence of clinical relevance, dynamic testing of growth hormone (GH) deficiency was not available. Possible GH deficiency was defined as an IGF-1 level of ≥2 standard deviations (SD) below the sex- and age-dependent reference range. Hyponatremia was defined as a sodium concentration of <136 mmol/l. Recovery from pituitary deficiency was defined as a successful cessation of the specific hormonal suppletion, including normalization of biochemical parameters. Secondary (complete) empty sella was defined as loss of pituitary height of ≥ 33% with >50% of the sella filled with cerebrospinal fluid and pituitary thickness ≤2 mm (14).

Treatment of IR-hypophysitis was at the discretion of the physician and in accordance to the European oncology guideline (2017) (15). In this guideline high dose corticosteroids (prednisolone 0.5–1 mg/kg) were advised in case of headache and hormone replacement therapy was advised in asymptomatic patients or in case of vague symptoms (e.g. mild fatigue or anorexia) without headache.

The data were analyzed using SPSS version 25.0. Descriptive statistics were expressed as absolute numbers, percentages, median with ranges or means with standard deviation (SD) depending on data distribution. The Chi-square and Fisher exact tests were used to compare categorical data and p-values of ≤ 0.05 were considered to indicate statistical significance. Differences between continue data with a skewed distribution were examined using the Kruskal Wallis and Mann Whitney U tests.

## Results

In this study 67 patients with IR-hypophysitis were included. Baseline characteristics and the clinical presentation of IR-hypophysitis are describes in **Table 1**. Most patients were male (63%), the mean age at diagnosis of IR-hypophysitis was 60.4 years (SD 11.3). The median time between the start of ICI to the diagnosis of IR-hypophysitis was 17 weeks (range 2–176). The median follow-up time from IR-hypophysitis to the end of the study period was 73 weeks (range 3–100). Patients had been treated for different types of cancer: 42 (63%) for melanoma, 6 (9%) for renal cancer, 5 (7%) for lung cancer and 14 (21%) for other types of cancers. 37% of patients were treated with anti-PD-1 monotherapy (nivolumab, pembrolizumab), 3% with anti-PD-L1 monotherapy (atezolizumab), 9% with anti-CTLA-4 monotherapy (ipilimumab, tremelimumab) and 51% with anti-CTLA-4/PD-1 combination therapy (ipilimumab, nivolumab).

**Table 1:** Patient characteristics of included patients.

Patients’ characteristics (n= 67)	
Mean age Sex *(% male)*	60.4 years (SD 11.3) 63% male
Type of cancer	
Melanoma Renal cancer Lung cancer Other malignancies*	63% (42 out 67) 9% (6 out 67) 7% (5 out 67) 21% (14 out 67)
Type of ICIs	
Anti-PD-(L)1 monotherapy Anti-CTLA-4 monotherapy Anti-CTLA-4/PD-1 therapy	40% (27 out 67) 9% (6 out 67) 51% (34 out 67)
Median time to IR-hypophysitis from start ICIs *(weeks)*	17 (range 2–176 weeks)
Symptoms at presentation	
Malaise and/or fatigue Nausea and/or anorexia Dizziness Muscle complains Headache Visual symptoms (no chiasm compression)	93% (41 out 44) 83% (45 out 54) 50% (14 out 28) 78% (18 out 23) 40% (26 out 65) 5% (3 out 65)
Pituitary insufficiency at diagnosis	
ACTH deficiency TSH deficiency LH/FSH deficiency IGF-1 level below ≤2 SD AVP deficiency	97% (65 out 67) 31% (21 out 67) 36% (18 out 50) 13% (4 out 30) 0% (0 out 67)
Hyponatremia	33% (22 out 67)
Signs of hypophysitis on MRI* Secondary empty sella during follow up	38% (9 out 24) 22% (7 out of 32)
Treatment*	
Low dose corticosteroids Medium dose corticosteroids High dose corticosteroids	27% (18 out 67) 57% (38 out 67) 16% (11 out 67)
Recovery of pituitary insufficiency at follow up	
Median follow up time *(weeks)* Recovery ACTH deficiency Recovery TSH deficiency Recovery LH/FSH deficiency	73 (range 3–495 weeks) 0% (27 out 27) 92% (12 out 13) 70% (7 out 10)

Percentages (number of total), Standard deviation (SD).

*Signs of hypophysitis on MRI: pituitary enlargement and/or enhancement of pituitary after gadolinium and/or increased stalk thickness.

*Low dose corticosteroids <40 mg hydrocortisone or equivalent per day versus medium dose ≥40 mg hydrocortisone or equivalent per day per day versus high dose ≥160 mg hydrocortisone or equivalent day.

*Other malignancies: advanced ovarian carcinoma, cervix carcinoma, laryngeal carcinoma, duodenal carcinoma, glioblastoma multiforme, Merckel cell carcinoma, breast cancer.

Clinical symptoms of IR-hypophysitis were non-specific in most cases: malaise or fatigue was the most reported symptom (93%), followed by nausea or anorexia (83%), muscle complaints such as pain or weakness (78%) and dizziness (50%). Local symptoms such as headaches and visual symptoms were present in 40% and 5% of patients, respectively.

Biochemically, ACTH deficiency was present in 97% of patients, with a median serum cortisol concentration of 40 nmol/L (reference range >130 nmol/L). Functional testing for ACTH deficiency with either a synacthen test or metyrapone test was only indicated in two patients confirming ACTH deficiency. TSH deficiency was present in 31% of patients at any point in time, LH/FSH deficiency in 36% of patients and an IGF-level of ≥ 2 SD below the reference range in 13% of patients. None of the patients developed arginine vasopressin (AVP) deficiency, formerly known as diabetes insipidus. Hyponatremia was present in 33% of patients. The presence of hyponatremia was not related to the presence of cerebral metastasis. No correlation was found between the sodium concentration and cortisol level or FT4. A typical MR image of hypophysitis (See **Figure 1** and Image 1) was present at diagnosis in 38% (9 out 24) of patients.

The treatment strategy for IR-hypophysitis was at the treating physician’s discretion, also depending on other concurrent irAEs. Low dose corticosteroids were defined as ≤ 40 mg, medium dose defined as >40 mg and high dose defined (HD) as ≥160 mg hydrocortisone or an equivalent dosage of corticosteroids per day. Patients with monotherapy anti-CTLA-4 induced hypophysitis were more frequently treated with HD corticosteroids. Most patients (73%) were initially treated with medium dose or HD corticosteroids. The corticosteroids were tapered to replacement therapy over a period of days to weeks.

During follow-up, recovery from TSH deficiency and LH/FSH deficiency was described in 92% and 70% of patients, respectively. The dose of corticosteroids) did not affect these recovery rates. The timing of recovery from TSH deficiency and LH/FSH deficiency could only be assessed in the patients without initiation of thyroid hormone (n=6) or testosterone (n=5). The mean concentration of thyroid hormone and testosterone at diagnosis did not differ between patients with or without hormonal suppletion. Recovery from TSH deficiency in these 6 patients was seen after a mean period of 14 days (range 7–28 days), while recovery from LH/FSH deficiency was seen after a mean period of 104 days (range 47–178 days). ACTH deficiency was reassessed in 27 out of 67 patients by cessation of corticosteroids and evaluating the cortisol level in the morning. However, none of the patients recovered from ACTH deficiency. Unfortunately, reassessment of (possible) GH deficiency during follow-up was not available.

In total 72% (48 out 67) of patients experienced other immune-related adverse events before or after the diagnosis of IR-hypophysitis. The most common immune-related adverse events were primary hypothyroidism or thyroiditis (28%), dermatitis or vitiligo (25%), hepatitis (21%), pneumonitis (9%), nephritis (8%), colitis (6%) and uveitis (5%). Hepatitis was more prevalent in patients treated with anti-CTLA-4/PD-1 combination therapy.

### Clinical differences of IR-hypophysitis between ICIs

The clinical differences between the treatment groups are outlined in **Table 2**.

**Table 2:** Differences of IR-hypophysitis.

	Anti-PD-(L)1 monotherapy	Anti-CTLA-4 monotherapy	Anti-CTLA-4/PD-1 combination therapy	p-value*
Patients’ characteristics (n = 67)				
Number of patients Mean age *(years)* Sex *(% male)*	27 58.7 48%	6 58.5 83%	34 62.1 71%	0.14 0.47
Median time to IR-hypophysitis from start ICI *(weeks)*	***21.9***	***10.6***	***13.9***	***0.04***
Symptoms at presentation				
Malaise and/or fatigue Nausea and/or anorexia Dizziness Muscle complaints Headache Visual symptoms	89% (16 out 18) 91% (19 out 21) 50% (8 out 16) 87% (13 out 15) **8% (2 out 26)** 0% (0 out 26)	100% (6 out 6) 83% (5 out 6) 50% (3 out 6) *No data* **83% (5 out 6)** 0% (0 out 6)	96% (22 out 23) 78% (21 out 27) 50% (4 out 8) 63% (5 out 8) **58% (19 out 33)** 9% (3 out 33)	0.66 0.59 1.0 0.21 **0.00** 0.44
Deficiencies at diagnosis				
ACTH deficiency TSH deficiency LH/FSH deficiency IGF-1 level below ≤2 SD Hyponatremia	100% (27 out 27) **11% (3 out 27)** **10% (2 out 22)** 0% (0 out 14) 19% (5 out 27)	100% (6 out 6) **67% (4 out 6)** **83% (5 out 6)** 0% (0 out 2) 33% (2 out 6)	94% (32 out 34) **41% (14 out 34)** **48% (11 out 23)** 29% (4 out 14) 44% (15 out 34)	0.58 **0.04** **0.00** 0.22 0.09
Laboratory results				
ACTH median (range) *Reference range 4–60 ng/L* Cortisol median (range) *Reference range 130–830 nmol/L* TSH median (range) *Reference range 0.35–5.0 mIU/L* FT4 median (range) *Reference range 10–22 pmol/L* LH median* (range) *Reference range 1–9 IU/L* FSH median* (range) *Reference range* Testosteron median* (range) *Reference range (10–31)* IGF-1 median standard deviation (range) Sodium median (range) *Reference range 135–145 mmol/L*	9 ng/L(2–25) 30 nmol/L (10–140) **2.4 mIU/L(0.07–63)** 13.0 pmol/L (6–21) **8.6 mIU/L (3.6–52)** **14.5 (2.8–31)** **15 nmol/L** **(range 3.6–36)** 0.3 SD (range -1.7–3.0) 138 mmol/L (range 118–140)	28.5 ng/L (22–35) 130 nmol/L (20–210) **0.14 (0.04–0.32)** 12 pmol/L (10–17) **1.0 mIU/L (0.86–4.5)5.8** (**1.9–11**) **0.58 nmol/L (range 0.5–3.2)** 2.7 SD (2.3–3.1) 136 mmol/L (range 126–139)	6 ng/L (2–46) 45 nmol/L (20–250) **1.2 mIU/L (0.01–18)** 12 pmol/L (7–22) **2.8 IU/L (0.2–8.9)** **5.4 (2.4–20)** **4.5 nmol/L (range 0.5–27)** -0.1 SD (-2.9–2.7 136 mmol/L (range 122–145)	0.13 0.09 **0.001** 0.49 **<0.001** **0.004** **0.01** 0.13 0.12
Signs of hypophysitis on MRI*	**0% (0 out 8)**	**60% (3 out 5)**	**55% (6 out 11)**	**0.02**
Secondary empty sella during follow up	0% (0 out 10)	0% (0 out 2)	22% (7 out 20)	0.11
Treatment*				
Low dose corticosteroids Medium dose corticosteroids High dose corticosteroids	**22% (6 out 27)** 70% (19 out 27) 7% (2 out 27)	**0% (0 out 6)** 17% (1 out 6) 83% (5 out 6)	**35% (12 out 34)** 53% (18 out 34) 12% (4 out 34)	**0.00**

Percentages (number of total).

* p value for chi-square or Fisher’s exact tests (in case the assumptions for chi-square test were violated). p- value for Kruskal Wallis tests for continues data.

*LH, FSH, testosterone in male patients.

* Signs of hypophysitis on MRI: pituitary enlargement and/or enhancement of pituitary after gadolinium and/or increased stalk thickness.

* Low dose corticosteroids <40 mg hydrocortisone or equivalent per day versus medium dose ≥40 mg hydrocortisone or equivalent per day per day versus high dose ≥160 mg hydrocortisone or equivalent day.

Bold font indicates statistical significance.

Differences were seen both in timing and in the degree of hypophysitis; anti-PD-(L)1 induced hypophysitis was seen after a median of 21.9 weeks versus after 10.6 and 13.9 weeks in patients with and CTLA-4 and anti-CTLA-4/PD-1 induced hypophysitis. Headache was mainly present in patients with anti-CTLA-4 and anti-CTLA-4/PD-1 induced hypophysitis (83% and 58% of patients, respectively versus 8% of patients with anti-PD-(L)-1 induced hypophysitis).

Differences in pituitary insufficiency were present between the treatment groups: TSH deficiency was seen in 11%, 67% and 41% and LH/FSH deficiency was seen in 10%, 83% and 48% of patients with anti-PD-(L)1 versus, anti-CTLA-4 and anti-CTLA-4/PD-1-induced hypophysitis, respectively. We analyzed the levels of LH, FSH and testosterone in male patients because 92% of female patients were postmenopausal. In accordance to the prevalence of hormone deficiencies, the median levels of TSH, LH, FSH and testosterone were significant lower in patients treated with anti-CTLA-4 and anti-CTLA-4/PD-1-therapy No differences were seen in prevalence of ACTH deficiency, hyponatremia and IGF-1 level ≥2 SD below the reference range. Also no differences were seen in the median levels of ACTH, cortisol, sodium and IGF-SD between the different treatment groups.

MRI abnormalities at diagnosis were seen in 56% (9 out 16) patients with anti-CTLA and anti-CLTA-4/PD-1 induced hypophysitis. Secondary empty sella during follow-up was evaluated by MRI in 32 patients after a median time of 28 months (range 4 to 94). In 22% (7 out 32) of patients secondary empty sella was seen on MRI. Secondary empty sella was only seen in patients treated with anti-CTLA-4/PD-1 combination therapy. Unfortunately, only in 3 of the 7 patients with secondary empty sella a diagnostic MRI at presentation had been performed, which showed signs of hypophysitis in all 3 patients (Image 1). In 9 out of 25 patients without secondary empty sella during follow up, an MRI at initial diagnosis of IR hypophysitis was available. Four of these 9 patients had radiological features of hypophysitis at diagnosis. Radiological features of hypophysitis at initial diagnosis were thus not related to secondary empty sella (p = 0.16).

## Discussion

In this cohort of patients with IR-hypophysitis, we could confirm clinically relevant differences between anti-PD-(L)1 versus anti-CTLA-4 mono- or combination therapy induced hypophysitis. The time to develop IR-hypophysitis seems to be shorter after anti-CTLA-4 mono- or combination therapy when compared to anti-PD-(L)1 monotherapy (median 10.6 and 13.9 versus 21.9 weeks, respectively), as was shown previously for other irAEs (16). Headache was more frequent in patients treated with anti-CTLA-4 mono- or combination therapy. MRI abnormalities (signs of hypophysitis or empty sella syndrome) were only seen in patients treated with anti-CTLA-4 mono- or combination therapy. Last, anti-CTLA-4 mono- or combination therapy induced more severe hypopituitarism with, in addition to ACTH deficiency, also TSH and LH/FSH deficiencies. Recovery from TSH and LH/FSH deficiency occurred in the majority of patients. ACTH deficiency, however, seems to be permanent.

Our study provides new data on the course and severity of anti-PD-(L)1 monotherapy versus anti-CTLA-4- but also anti-CTLA-4/PD-1 combination therapy induced hypophysitis. Most studies have described IR-hypophysitis caused by either anti-CLTA-4 or PD-(L)1 monotherapy (9, 10, 17–19). In a 10-year assessment by Di Dalmazi et al. differences between IR-hypophysitis caused by anti-CTLA-4 versus anti-PD-(L)1 monotherapy are described, but the number of patients with IR-hypophysitis caused by anti-CTLA-4/PD-1 combination therapy was too small to analyze, which was the reason for us to perform this study (9). A recent study performed by Jessel et al. described the differences between anti-CTLA-4/PD-1 induced hypophysitis (n = 53) versus anti-PD-(L)1 induced hypophysitis (n = 13) (19). Our study results are in accordance with their results, with shorter latency time in patients with anti-CTLA-4/PD-1 therapy, higher prevalence of headache in anti-CTLA-4/PD-1-induced hypophysitis, and more frequent TSH deficiency and LH/FSH deficiency in anti-CTLA-4/PD-1 induced hypophysitis compared to anti-PD-(L)1 monotherapy induced hypophysitis. However, in contrast to our findings, with no MRI abnormalities in patients with anti-PD(L1) induced hypophysitis, Jessel et al. found MRI abnormalities in 1 (out of 5) patient with anti-PD-(L)1-induced hypophysitis.

In the study of Dalmazi et al. differences between monotherapy with anti-PD-1 and monotherapy with anti-CTA-4 were analyzed (9). Comparable to our study pituitary deficiencies were seen more frequently after anti-CTLA-4 versus anti-PD-(L)1 therapy. The same authors found a higher prevalence of hyponatremia in anti-PD-(L1) versus anti-CTLA-4 induced hypophysitis (62% versus 39%, respectively). The authors hypothesized that this difference could be related to more severe ACTH deficiency in anti-PD-(L)1-induced hypophysitis. However, we did not find a difference in the median sodium level or the prevalence of hyponatremia between PD-(L)1 versus CTLA-4 mono- or combination therapy induced hypophysiits. Moreover, no correlation was found between the cortisol and sodium level, which does not support the hypothesis of Dalmazi et al. (9).

In our cohort, none of the patients developed AVP-deficiency and this seems very rare in IR-hypophysitis (10, 17, 18, 20), although Dalmazi et al. reported AVP deficiency in 2–3% of patients (9). In our opinion, AVP deficiency should raise the suspicion for pituitary metastasis and is an indication for imaging.

In our study, recovery from TSH and LH/FSH deficiency was found in the far majority of tested patients (92% and 70%, respectively). Nguyen et al. (21) described 62 patients with IR-hypophysitis and reported a recovery rate of TSH, LH/FSH and ACTH deficiency of respectively 24%, 58% and 0% (using comparable definitions for TSH deficiency and recovery time). The difference in recovery rate of TSH deficiency between the studies might be explained by a more severe TSH deficiency in patients with anti-CTLA-4 induced hypophysitis; 57 of 62 patients in the study of Nguyen were treated with anti-CTLA-4 therapy (21). Currently, no guidelines exist regarding re-evaluation of TSH or LH/FSH deficiency. Reevaluation of pituitary deficiencies is dependent on the preference of the physician as well as the patient’s clinical condition and prognosis, which may explain the wide range in recovery rates of TSH and LH/FSH deficiency reported in literature. Recovery from ACTH deficiency was not seen in our cohort, which is in line with previous reports (10, 17–21).

The exact mechanism underlying IR-hypophysitis is still largely unknown. Interestingly, ACTH deficiency is prominent in IR-hypophysitis. However, in other pituitary disorders including acquired forms after radiation treatment, the secretion of GH and TSH are most vulnerable and are frequently affected before ACTH or LH/FSH deficiency develops (22, 23). Because anti-PD-(L)1 induced hypophysitis primarily causes ACTH deficiency and anti-CTLA-4 therapy is associated with more pronounced hypopituitarism with involvement of plural pituitary axes, it is hypothesized that the pathophysiological mechanism of IR-hypophysitis differs between the two. The checkpoint CTLA-4 is not only expressed on T-cells but also on pituitary cells. The degree of CTLA-4 expression in the adenohypophyses varies greatly between individuals, possibly explaining why some patients develop IR-hypophysitis and others do not. Complement deposition as well as infiltration of lymphocytes was seen in the pituitary tissue of mice injected with ipilimumab and in an autopsy report of a patient with IR-hypophysitis (24, 25). CTLA-4 is an IgG1 monoclonal antibody which can bind and activate the complement cascade, leading to a type 2 hypersensitivity reaction. It is postulated that this type-2 reaction triggers the adaptive immune system, leading to a type-4 hypersensitivity reaction. This type-4 hypersensitivity reaction is more classical for autoimmune disorders. Although pituitary antibodies were detected by indirect immunofluorescence in one study, the exact pituitary antigens are not yet identified (26).

Much less is known about the pathophysiological mechanism of anti-PD-(L)1-induced hypophysitis. Nivolumab, an IgG4 monoclonal antibody, cannot activate the complement cascade. Maybe the pathogenesis of anti-PD-(L)1-induced hypophysitis resembles IgG-4 related hypophysitis. Because of the predominance of ACTH deficiency in anti-PD-(L)1-induced hypophysitis, it has also been postulated that ACTH secreting cells express the highest levels of PD-1 (7, 9). In the first autopsy study of a patient with anti-PD-1-induced hypophysitis, lymphocyte infiltrates were found in the anterior lobe of the pituitary and the number of ACTH cells was reduced (27). Another study analyzed pituitary antibodies in patients with IR-hypophysitis and found anti-corticotroph antibodies In 10% (2 out 20) of patients (28). Interestingly, these two patients also exhibited ectopic ACTH expression in the tumor. Ectopic ACTH expression is reported previously in various cancers. In the patients without ectopic ACTH expression in the tumor no anti-corticotroph antibodies have been detected. It was hypothesized that ACTH expression in tumors may evoke autoreactive T cells with specific injury to the corticotroph cells, possibly explaining the predominance of ACTH deficiency in IR-hypophysitis.

Interestingly, the current guidelines for treatment of IR-hypophysitis differ significantly between endocrinologists and oncologists (**Supplementary Material**) (5, 6). The guideline of the European Society of Endocrinology is restrictive in corticosteroid use and the need for MRI or visual field examination in comparison to the guideline of The European Society for Medical Oncology. Because of the clinical differences such as headache and MRI abnormalities in patients with anti-PD-(L)1 monotherapy versus anti-CTLA-4 (mono- or combination) therapy, it may be questioned whether the treatment strategy should be similar (9, 19). Percik et al. suggested to classify separate forms of hypophysitis: IR-hypophysitis should be reserved to describe the symptomatic phase of hypophysitis with headache and imaging suggestive of hypophysitis while isolated ACTH deficiency should be used in PD-(L)1 induced hypophysitis without local symptoms and without other pituitary dysfunction or radiological evidence of hypophysitis. IR-hypopituitarism can be used to describe the long-term deficiencies of at least two pituitary hormones. This new nomenclature can facilitate different treatment strategies (29). Our results underscore the use of this new nomenclature.

Corticosteroids are the cornerstone in the treatment of IR-hypophysitis. However, there is still debate upon the optimal dose of corticosteroids. Endocrine irAEs are associated with an improved overall and progression-free survival, probably because eIRAEs are a marker of immune response and thus of anticancer effects in patients treated with ICIs (30–32). HD corticosteroids do not seem to affect the recovery rate of hypopituitarism (1–19–21). Importantly, the use of HD glucocorticoids in patients with anti-CTLA-4-induced hypophysitis was reported to affect overall survival negatively (10). Unfortunately, we could not analyze the effect of corticosteroids dosage on survival because of the heterogeneity of our cohort with patients treated adjuvant or in palliative setting. In our opinion, because of the potential negative impact of HD corticosteroids on overall survival and the lack of expected benefits, in accordance with the ESE guideline (6) (**Table 3**), HD corticosteroids should be used in case of IR-hypophysitis plus chiasm compression or severe headache (after exclusion of pituitary metastasis). In case of adrenal crisis, treatment with intravenous or intramuscular hydrocortisone is indicated. Suppletion of thyroid hormones or sex hormones should be initiated on biochemical and clinical grounds. If biochemical aberrations are small without clinical signs of hormonal insufficiency, monitoring for spontaneous recovery could be an alternative strategy. If thyroid hormones or sex hormones are started, and the clinical condition and prognosis of the patient are positive, reevaluation of TSH and LH/FSH deficiency may be considered after 3 months. Reassessment of ACTH deficiency is not recommended, given the virtually absent recovery from ACTH deficiency in the literature (9, 10, 17–20).

**Table 3:** Treatment guideline for IR-hypophysitis according to European Society of Endocrinology.

IR-hypophysitis In case of IR-hypophysitis with adrenal crisis In case of IR-hypophysitis with optic chiasm compression or severe untreatable headache	Hydrocortisone 15–25 mg, 2–3 times daily Hydrocortisone 100 mg intravenously or intramuscularly followed by 50 mg every 6 hours High dose corticosteroids
All patients should be trained to adjust hydrocortisone dose in case of stress or illness.	
Treatment with LT4 if FT4 is decreases or low normal, consider tapering and stopping to assess for recovery during follow up. Glucocorticoids should be started before the initiation of thyroid hormone replacement. Start suppletion of gonadal hormones in case of persistent hypogonadism.	

Some limitations must be considered when interpreting our results. The retrospective nature of this study and the small number of patients with monotherapy CTLA-4 indicate the necessity to confirm our results in larger cohorts. Furthermore, we could not calculate the incidence of IR-hypophysitis due to the lack of information on the total number of patients treated with ICIs in this period. Unfortunately, the timing of recovery from TSH and LH/FSH deficiency was challenging to assess because of its dependency on the physician’s evaluation strategy. Despite these limitations, we were able to evaluate the long-term follow-up of a large cohort of patients with IR-hypophysitis and to provide new data on the clinical differences between anti-PD-(L)1 and anti-CTLA-4 (mono and combination) therapy induced hypophysitis.

## Conclusion

IR-hypophysitis is a common eIRAE of treatment with ICIs. Anti-PD-(L)1 induced hypophysitis differs from anti-CTLA-4 mono- or combination induced hypophysitis with regard to clinical symptoms, MRI abnormalities and the degree of hypopituitarism, which is more pronounced after anti-CTLA-4 mono- or combination therapy. Although ACTH deficiency seems to be permanent, recovery from LH/FSH and TSH deficiency may be expected in the majority of patients.

## Data availability statement

The raw data supporting the conclusions of this article will be made available by the authors, without undue reservation.

## Ethics statement

The studies involving humans were approved by Medische ethische toetsings commissie UMC Utrecht. The studies were conducted in accordance with the local legislation and institutional requirements. The human samples used in this study were acquired from a by- product of routine care or industry. Written informed consent for participation was not required from the participants or the participants’ legal guardians/next of kin in accordance with the national legislation and institutional requirements.

## Author contributions

SL: Formal Analysis, Investigation, Methodology, Resources, Writing – original draft, Writing – review & editing. KS: Writing – review & editing. MD: Writing – review & editing. GV: Writing – review & editing. JD: Investigation, Writing – review & editing. HS: Supervision, Writing – review & editing.
