# Supplementary material for: Differences in checkpoint-inhibitor-induced hypophysitis: mono- versus combination therapy induced hypophysitis
Source: Front Endocrinol (Lausanne). 2024 Jul 29;15:1400841. doi: 10.3389/fendo.2024.1400841 (PMC11317883; doi:10.3389/fendo.2024.1400841)
Supplement: Supplementary file 1 [file Table_1.docx]

Supplementary Material

**Differences in checkpoint-inhibitor-induced hypophysitis**

Stephanie van der Leij^1,2^, Karijn P.M. Suijkerbuijk^3^, Medard F.M. van den Broek^1,3^, Gerlof D. Valk^1,3^, J.W. Dankbaar^4^, Hanneke M van Santen^2,5^

**Correspondence:** Stephanie van der Leij

[s.m.vanderleij@umcutrecht.nl](mailto:s.m.vanderleij@umcutrecht.nl)

**Supplementary material.** European guidelines for IR-hypophysitis

| Management IR-hypophysitis | ESE guideline^6*^ | ESMO guideline^9*^ |
| --- | --- | --- |
| Hypophysitis grade 1-2  *(Asymptomatic or vague symptoms, no headache, no electrolyte disturbance)* | Hydrocortisone 15-25 mg, 2-3 times daily  **Treatment with LT4 if FT4 is decreases or low normal* | Continue ICIs  Hydrocortisone 20-10 mg  MRI pituitary  **Consider replacement of LT4 based on symptoms (if TSH ± FT4 is low)* |
| Hypophysitis grade 2  *(Symptoms of headache, no visual disturbance)* | Hydrocortisone 15-25 mg, 2-3 times daily  **Treatment with LT4 if FT4 is decreases or low normal* | Withhold ICIs  Prednisone 0.5-1.0 mg/kg, wean corticosteroids to 5 mg prednisone over 1-2 weeks  MRI pituitary if headache is present & visual field assessment  **Consider replacement of LT4 based on symptoms (if TSH ± FT4 is low)* |
| Hypophysitis grade 3-4  (Severe mass effect symptoms of severe hypocortisolism, hypotension or severe electrolyte disturbance) | In case of adrenal crisis hydrocortisone 100 mg i.v. or i.m. followed by 50 mg/6 hours  In case of chiasm compression or severe headache high dose corticosteroids  **Treatment with LT4 if FT4 is decreases or low normal* | Withhold ICIs  (Methyl)prednisolone 1 mg/kg i.v., wean corticosteroids to 5 mg over 2-4 weeks.  MRI pituitary & consider visual field assessment  **Consider replacement of LT4 based on symptoms (if TSH ± FT4 is low)* |
| **ESE: European Society of Endocrinology. *ESMO: European Society for Medical Oncology*  **Glucocorticoids should be started before the initiation of thyroid hormone replacement*  *Consider tapering levothyroxine (LT4) replacement periodically in patients with low dose replacement to assess recovery*  *All patients should be trained on stress dosing “sick day rules”* | | |
